# On the Effect of the Co-Introduction of Al and Ga Impurities on the Electrical Performance of Transparent Conductive ZnO-Based Thin Films

**DOI:** 10.3390/ma15175862

**Published:** 2022-08-25

**Authors:** Abil S. Asvarov, Aslan K. Abduev, Akhmed K. Akhmedov, Vladimir M. Kanevsky

**Affiliations:** 1 Institute of Physics, Dagestan Research Center of Russian Academy Sciences, Yaragskogo Str., 94, 367015 Makhachkala, Russia; 2 Shubnikov Institute of Crystallography, Federal Scientific Research Center “Crystallography and Photonics” of Russian Academy of Sciences, Leninsky Prospect, 59, 119333 Moscow, Russia; 3 Basic Department of Nanotechnology and Microsystem Technology, Academy of Engineering, RUDN University, 117198 Moscow, Russia

**Keywords:** ZnO, TCO, thin film, magnetron sputtering, ceramic target, doping, Al, Ga, resistance, nanocrystallite

## Abstract

In this study, a set of ZnO-based thin films were prepared on glass substrates at various substrate temperatures via the direct current magnetron sputtering of ceramic targets with the following compositions: pure ZnO, Al-doped ZnO with doping levels of 1 and 2 at.%, Ga-doped ZnO with doping levels of 1 and 2 at.%, and (Al, Ga)-co-doped ZnO with doping levels of 1 and 2 at.% for each impurity metal. The dependencies of sheet resistance, carrier concentration, and Hall mobility on the substrate temperature were studied for the deposited films. The results of evaluating the electrical performances of the films were compared with the data of their XRD study. According to the XRD data, among all the deposited ZnO films, the maximum crystallinity was found in the co-doped thin film with doping levels of 2 at.% for each impurity metal, deposited at a substrate temperature of 300 °C. It was revealed that the observed increase in the Hall mobility and carrier concentration for the co-doped films may, in particular, be due to the difference in the preferred localization of Ga and Al impurities in the ZnO film: the Ga ions were mainly incorporated into the crystal lattice of ZnO nanocrystallites, while the Al impurity was mostly localized in the intercrystalline space at the grain boundaries.

## 1. Introduction

A thin-film transparent electrode is an integral passive element of many optoelectronic devices, such as liquid crystal displays, solar cells, light-emitting diodes, radiation and gas resistive-type sensors, etc. [1,2,3,4]. From the term “transparent electrode”, it follows that the thin films of this functional application must be characterized by high optical transmittance in the visible region and have as little electrical resistivity as possible. Transparent conductive oxide (TCO) thin films based on wide-gap oxides (In_2_O_3_, ZnO, SnO_2_, etc.) are currently the most widely used materials for transparent electrodes. From the point of view of the combination of electrical and optical characteristics, indium tin oxide (ITO) thin films have long been the main industrial material for transparent electrodes. However, the inadequate indium mine volumes and the associated high cost of ITO make it urgent to search for other transparent conductive materials with optical and electrical characteristics acceptable to device manufacturers. One of the main alternative TCO materials is thin films based on ZnO doped with elements of the III group of the periodic table (B, Al, Ga, In). Doped-ZnO TCO thin films are already widely used as transparent electrodes in thin-film solar cells, as well as in low-emission and antistatic functional coatings [5,6,7,8,9]. However, for the wider use of the ZnO-based TCO films as transparent electrodes in modern optoelectronic devices, some improvements in thin films’ performance regarding their electrical properties and long-term stability are further required both by optimizing the thin-film deposition processes and by tuning the film doping. Regarding the doping optimization, if a large number of earlier studies were devoted to finding the best donor for ZnO TCO films, as well as optimizing the level of doping, an increasing number of reports have recently appeared indicating that the simultaneous co-doping of ZnO with two or more types of impurities leads to a noticeable improvement in the electrical characteristics of TCO films, as well as their microstructures [10,11,12,13,14,15]. In particular, we have shown that the co-doping of ZnO with Ga and B leads to the formation of denser films with an improved film microstructure and, as a result, to an increase in the thermal stability of ZnO TCO films [10]. In turn, Vorobyeva et al., on the basis of a comparative study of the effect of indium and gallium impurities on the properties of spin-coated ZnO films, showed that the case of In, Ga co-doped ZnO is preferable to the ones of ZnO doped with Ga or In [10]. It has been hypothesized that the observed improvement in electrical conductivity is caused by the increase in donor elements’ solubility in the ZnO structure for co-doped films, compared with single-doped ZnO, due to the decrease in ZnO crystal lattice strains. In general, based on the analysis of a large number of similar studies, it can be concluded that co-doping is one of the most promising ways to solve the urgent problem of improving the electrical characteristics of ZnO-based TCO films formed by various types of physical and chemical deposition methods (e.g., magnetron sputtering, laser ablating, chemical vapor deposition, spin coating, etc.) [16,17,18,19,20,21].

In the present work, using the examples of pure ZnO, Ga-doped ZnO, Al-doped ZnO, and Ga, Al-co-doped ZnO thin films deposited via direct current (dc) magnetron sputtering at various substrate temperatures, the influence of the doping type (single doping or co-doping), as well as of the total doping level, on the microstructure and functional properties of ZnO-based TCO thin films was evaluated. The results revealed that, by increasing the substrate temperature up to 300 °C, the co-doping of ZnO allowed us to obtain highly crystalline ZnO TCO thin films with strong textures along the (002) directions, as well as with improved electrical performance. In this paper, the obtained results for the studied metal oxide thin-film system are discussed based on the relative position of the host (Zn) and impurity (Ga and Al) atoms on the metal reactivity series. In our opinion, the presented findings can be useful for further developments in materials engineering in the area of transparent electronics.

## 2. Materials and Methods

Briefly, 150 nm thick ZnO-based TCO thin films at various substrate temperatures were deposited on glass substrates via sputtering using a homemade direct current magnetron sputtering system equipped with a multipositional drum-type assembly in a single vacuum cycle [22]. The sputtering targets used for deposition were pure ZnO, ZnO single-doped with 1 and 2 at.% Al, ZnO single-doped with 1 and 2 at.% Ga, and co-doped ZnO containing 1 and 2 at% of each of the Al and Ga impurity ceramic discs with a diameter of 51 mm. The abbreviations of the sputtered films used further in the article, depending on the impurity content in the sputtered target on the basis of zinc oxide, are given in Table 1. 

For each target, four thin-film samples were deposited in a single vacuum cycle at the substrate temperatures of 50, 100, 200, and 300 °C under the following deposition conditions: Ar working pressure of 0.5 Pa, discharge current of 270 mA, and discharge voltage of 720 V. The film thickness was controlled in situ using a quartz microbalance.

The crystalline structure and phase of the ZnO-based TCO films deposited at various substrate temperatures on glass substrates were measured using an X-ray diffractometer (XRD, X’PERT PRO MPD, Malvern Panalytical Ltd., Malvern, UK) with a CuKα source (λ = 1.5418 Å) and power of 40 kV/30 mA. The morphology of the thin films on Si substrates was studied via scanning electron microscopy (SEM, Leo-1450, Carl Zeiss, Oberkochen, Germany). In addition, the cross-sectional SEM images of the deposited films were used to confirm whether their thicknesses matched the initially required value (150 nm). The sheet resistance of the thin films was measured using a four-point technique (IUS-3, Moscow, Russia). The optical transmittance of the thin films coated on glass substrates was recorded using an optical spectrophotometer (Shimadzu UV-3600, Tokyo, Japan).

## 3. Results

**Figure 1:**
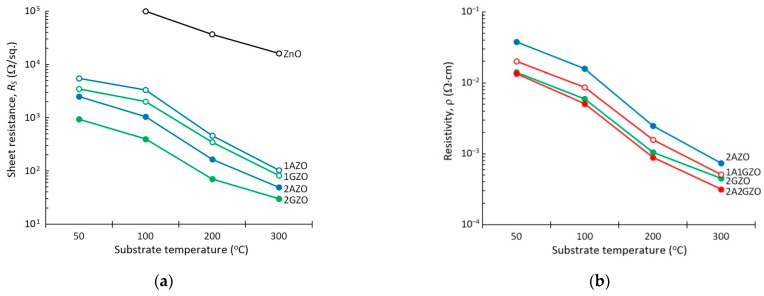
(**a**) Sheet resistance of pure ZnO and ZnO thin films single-doped with Al or Ga (1AZO, 1GZO, 2AZO, and 2GZO) as a function of substrate temperature; (**b**) resistivity of single-doped (2AZO, 2GZO) and co-doped (1A1GZO and 2A2GZO) thin films as a function of substrate temperature.

The measurement data of the sheet resistance *R_S_* of the pure ZnO and single-doped thin films (1AZO, 2AZO, 1GZO, and 2GZO) as a function of the substrate temperature are shown in Figure 1a. The results confirmed the well-known fact that the doping of ZnO with donor-type impurities, such as Al or/and Ga, allows a decrease in the sheet resistance of the films by several orders of magnitude in comparison with that of pure ZnO and that an increase in the doping level in both the Ga- and Al-doped ZnO cases also leads to improvement in the electrical conductivity of these thin films. Figure 1 also demonstrates that, at the same doping levels, the sheet resistance of Ga-doped ZnO films was lower than that of the Al-doped ZnO over the entire substrate temperature range, i.e., under our synthesis conditions, Ga was a more successful and efficient donor than Al. This may be related to gallium’s advantages such as good lattice matching with the ZnO lattice, less reactive and more resistant to oxidation than Al elements, thus playing an important role both at the stage of the sintering of a ceramic target and at the stage of thin-film deposition from this target [13,23]. In turn, the dependence of the resistance of the single-doped films on the substrate temperature showed that, in order to meet the acceptable TCO resistance requirement (*R_S_* < 100 Ω/sq., which corresponds to a resistivity ρ less than 1.5 × 10^−3^ Ω·cm at a films thickness of 150 nm), it is necessary to carry out the deposition of ZnO-based TCO films with a doping level above 1 at.% at a substrate temperature close to 300 °C.

Figure 1b compares the dependences of resistivity on substrate temperatures for single-doped and co-doped ZnO thin films. It is remarkable that the following two rules hold true for the substrate temperature range under study:

- For film compositions with their total impurity content at 2% (2AZO, 2GZO, and 1A1GZO), at each substrate temperature, the values of the film resistivity of co-doped films were always less than those of the films single-doped with Al but greater than that of the case of single doping of ZnO with Ga, i.e., ρ_2GZO_ < ρ_1A1GZO_ < ρ_2GZO_;
- By comparison, the co-doped 2A2GZO thin films, in which Al and Ga were simultaneously present in equal amounts at the level of 2%, demonstrated the best values in terms of resistivity.

The resistivity of the co-doped 2A2GZO film became less than 1.0 × 10^−3^ Ω·cm already at a substrate temperature of 200 °C, and with a further increase in the substrate temperature, it reached a fairly low value of ρ = 3.2 × 10^−4^ Ω·cm. At the same time, the less attractive resistivity values of 7.4 × 10^−4^, 5.1 × 10^−4^, and 4.5 × 10^−4^ Ω·cm were achieved in the 2AZO, 1A1GZO, and 2GZO samples deposited at 300 °C, respectively.

Against the background of the already known fact that Ga is a more efficient donor for ZnO than Al [23,24,25], the first of the above regularities (ρ_2GZO_ < ρ_1A1GZO_ < ρ_2GZO_) seems logical. The second founded regularity (namely, that the simultaneous doping of ZnO with 2 at.% Al and 2 at.% Ga led to a noticeable decrease in resistivity compared with single-doped films), from our point of view, requires further research and correct interpretations, since doped-ZnO TCO thin films are usually characterized by the presence of an optimal impurity doping level in the region of 2–3 at.%, above which a deterioration in electrical properties is usually observed due to various impurity-induced distortions in the host ZnO microstructure [26,27,28,29].

**Figure 2:**
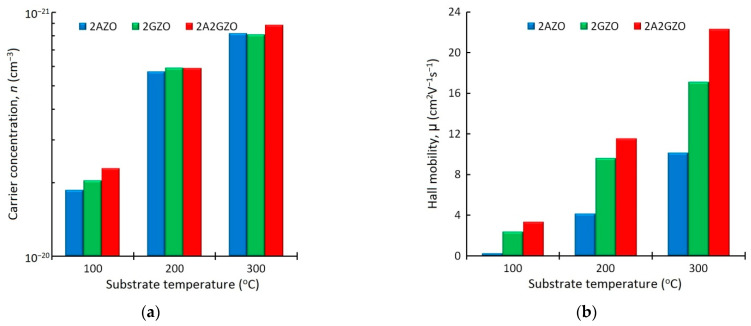
(**a**) Carrier concentration of single-doped 2AZO and 2GZO thin films and co-doped 2A2GZO thin films as a function of substrate temperature; (**b**) Hall mobility of single-doped 2AZO and 2GZO thin films and co-doped 2A2GZO thin films as a function of substrate temperature.

Figure 2 shows the electrical characteristics of the 2AZO, 2GZO, and 2A2GZO thin-film samples. In Figure 2, there is no region corresponding to 50 °C, due to the fact that these films had high values of resistivity, beyond the measurement limits of our Hall measurement setup. The results presented in Figure 2 showed that the regularities noted above were primarily determined by the nature of the influence of the substrate temperature on the Hall mobility of charge carriers. It can be seen that the carrier concentration *n* naturally increased with the increase in the substrate temperature (from ~2.0 × 10^20^ at 100 °C to ~8.5 × 10^20^ cm^−3^ at 300 °C). The results may be attributed to the fact that the dopants were effectively activated (Al^3+^ and/or Ga^3+^ substitute for Zn^2+^ sites) during film growth due to the higher kinetic energy of the adsorption atom. However, it can be seen that, at each substrate temperature, there was no significant difference in the values of the carrier concentration upon passing from one type of film to another.

At the same time, a much stronger dependence on the impurity composition in ZnO manifested itself when studying the Hall mobility μ of single-doped and co-doped thin films as a function of the substrate temperature (Figure 2b). In general, all three types of films were characterized by an increase in the Hall mobility with the increase in the substrate temperature, which is due to the increase in the kinetic energy of adatoms and the associated improvement in the crystalline quality of the ZnO films. It is much noteworthy that the co-doped 2A2GZO thin film, in which the total amount of the introduced impurities had a two-fold advantage, was characterized by a similar carrier concentration and significantly higher Hall mobility than single-doped GZO and AZO thin films. At the substrate temperature of 300 °C, μ reached 10.1, 17.1, and 22.3 cm^2^V^−1^s^−1^ for single-doped 2AZO, 2GZO, and co-doped 2A2GZO, respectively.

It should also be noted here that, as shown by spectrometric measurements, all the films deposited at a substrate temperature above 100 °C were characterized by a high average transmittance *T* > 85% in the visible range (as an example, the transmittance spectra of single- and co-doped thin films with ρ < 10^−3^ Ω·cm are shown in Figure S1 in the Supplementary Materials (SM)). In this case, the appearance of the spectrum next to the absorption edge depended not so much on the type of doping as on the substrate temperature and the total impurity (Al or/and Ga) content in the ZnO host, which correlated well with the data on measuring the carrier concentration *n* in these films, since the behavior of the film near the absorption edge is determined primarily by *n* [30,31].

Thus, in the course of a comparative study of the electrical characteristics of the single- and co-doped ZnO films deposited in our experiment, the following was found:

- Ga-doped ZnO thin films performed better than Al-doped ZnO thin films in terms of their resistivity and mobility;
- In order to achieve the maximum values of electrical performance in Ga-doped ZnO films, it was necessary to carry out sputtering at elevated substrate temperatures (close to 300 °C) and a doping level above 1%;
- The introduction of an additional amount of Al impurity into the ZnO film with 2 at.% Ga further reduced its resistivity, mainly due to an increase in mobility μ.

Here, it should be noted once again that, contrary to the expectation that doubling the total amount of introduced Al and Ga impurities should rather lead to an increase in the concentration *n*, we obtained the opposite result, namely, that the simultaneous co-introduction of 2 at.% Ga and 2 at.% Al in ZnO led to a noticeable increase in the mobility μ compared with the films single-doped with 2 at.% Al or 2 at.% Ga.

It is known that enhancing the mobility in polycrystalline thin films is often associated with an improvement in their morphology and crystallinity, diminishing defects in the lattice, or a reduction in the ionized impurity scattering inside the crystallites, thus forming a continuous film [12]. In order to gain more information about the aspects leading to the enhanced electrical performance of the co-doped 2A2GZO thin film, additional comparative investigations of the microstructure of single- and co-doped thin films deposited at the substrate temperature of 300 °C were performed.

The SEM studies by using an SEM Leo-1450 setup did not reveal any noticeable differences in the surface morphology of the 2AZO, 2GZO, and 2A2GZO thin films deposited at 300 °C (Figure S2 in SM). All the 150 nm thick samples, regardless of their doping type and level, exhibited a fairly smooth morphology with distinguishable lateral sizes of surface irregularities below 20 nm without any large inclusions, which is typical for nanocrystalline ZnO thin films with a doping level of 2% or more deposited at a moderate substrate temperature [32].

**Figure 3:**
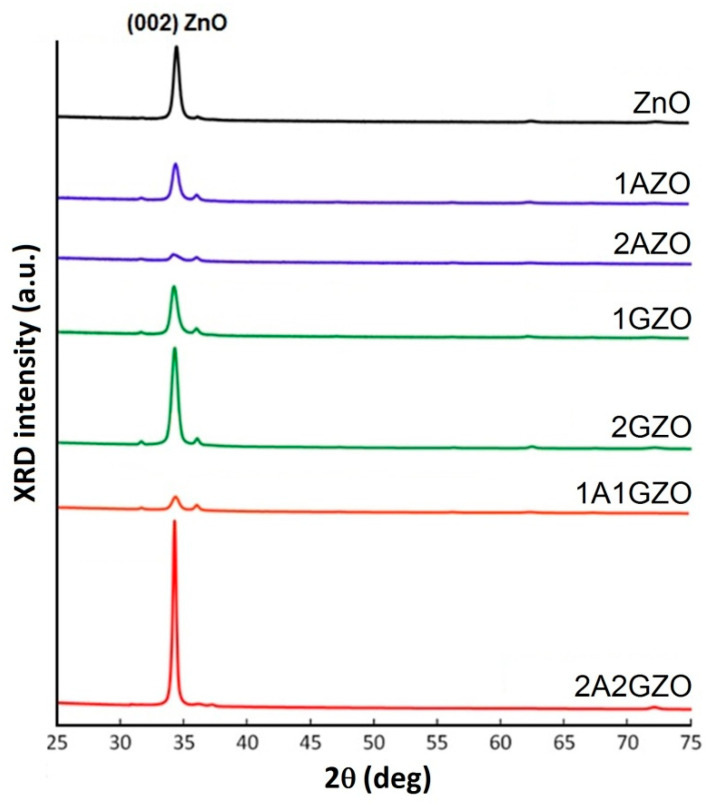
XRD spectra of pure ZnO, single-doped (1AZO, 2AZO, 1GZO, and 2GZO) and co-doped (1A1GZO and 2A2GZO) thin films deposited at substrate temperature 300 °C.

Figure 3 presents the XRD patterns of pure, single- and co-doped ZnO thin films deposited at the substrate temperature of 300 °C. All the films had a dominant (002) ZnO peak, indicating the typical hexagonal wurtzite structure of the ZnO material with a preferred orientation toward the polar c-axis of ZnO nanocrystallites normal to the film surface. The main characteristics of the dominant 002 peak depending on the type of doping and level of impurity content are summarized in Table 2.

From Figure 3 and Table 2, it can be seen that the appearance of XRD patterns significantly changed depending on the type and level of doping. From a comparison of the XRD spectra with each other, it can be seen that, with a two-fold increase in the amount of the introduced impurity(-ies), multidirectional tendencies emerged depending on the type of the introduced impurity.

In the case of single-doped ZnO films with Al, the peak was substantially suppressed and broadened with the increase in the doping level, which indicates a significant deterioration in the crystalline quality of the ZnO host under Al doping. This phenomenon agrees well with the results of previous works [33,34,35], in which it was noted that increasing the doping content above 1 at.% may result in interstitial positioning of Al atoms as well as the formation of oxide compounds into the noncrystalline region in the grain boundaries. At the same time, in the case of doping ZnO with gallium, with an increase in the doping level to 2 at.%, the XRD peak intensity became slightly higher than those of the pure ZnO film. This indicates that, at some concentrations, Ga impurity, unlike Al, does not suppress the crystallization of ZnO film; on the contrary, it can enhance the crystallization of the ZnO film [36].

However, the most remarkable thing occurred in the case of the simultaneous co-introduction of Al (an inhibitor of ZnO crystallization) and Ga (a promoter of ZnO crystallization) impurities in ZnO. In the 1A1GZO thin film, which was deposited from the target with total impurities content of 2 at.%, the intensity of the XRD peak occupied an intermediate value compared with the intensities of 2AZO and 2GZO samples deposited from the targets with the same impurity levels. At the same time, as shown in Figure 3, the (002) peak intensity drastically increased upon a further two-fold increase in the Al and Ga impurity content in the target (2A2GZO sample). In addition, the (002) peak integral breadth of this sample was remarkably narrower than that of the other samples (Table 2). This indicates that, with a certain combination of aluminum and gallium impurities, it is possible to achieve an even more significant enhancement in the crystallization processes of ZnO films via co-doping than those of single doping only with Ga. Similar results were noted in other studies [12,37]. Pham et al. [12] demonstrated that the presence of a small amount of In dopants (0.1 at%), combined with mainly Ga dopants (4.9 at.%) in the ZnO target, could significantly improve the crystallization of sputtered ZnO films. In its turn, Ebrahimifard et al. [37] observed that additional doping with Ga in Al-doped ZnO could reduce crystal deformation, resulting in higher crystallinity.

**Figure 4:**
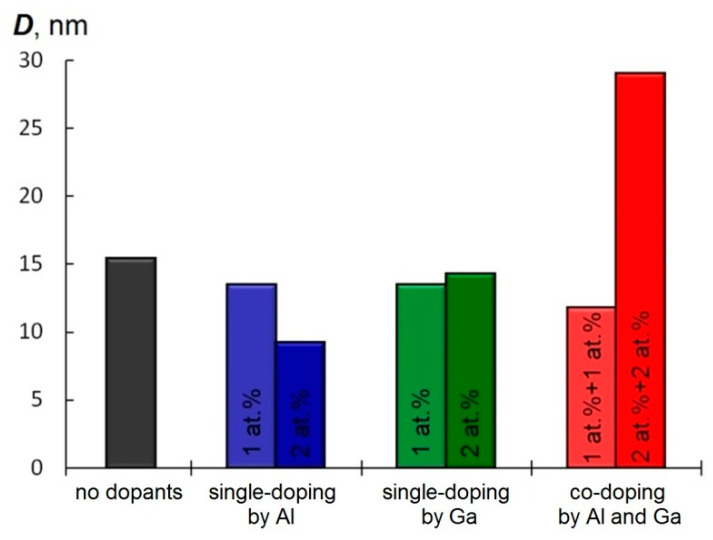
Average grain size of the deposited ZnO-based thin films deposited at substrate temperature 300 °C.

To assess the crystalline quality, the average crystallite sizes *D* of the film samples were estimated using Scherrer’s formula [38]. As seen in Figure 4, the crystallite size decreased from *D* = 15 nm in the pure ZnO to 10 nm upon introducing Al (up to 2 at.%) in ZnO. At the same time, there was no significant effect of the single doping of ZnO with Ga on the average crystallite size. Here, it is remarkable that the introduction of both elements in the amounts of 2 at% in the ZnO thin film (2A2GZO sample) allowed a significant increase in the average crystallite size *D* of up to ~30 nm, which was two times of magnitude higher than those of the pure ZnO.

Thus, on the whole, the data obtained during the XRD characterization of the microstructure of the deposited ZnO-based TCO thin films correlated well with our results measuring their electrical characteristics. 

The observed enhancement in mobility μ may be due to the improvement in the crystallinity of the ZnO film detected under the simultaneous introduction of Al and Ga impurities. Increasing the crystalline grain size *D* in the 2A2GZO sample, as proved in Figure 4, resulted in a decrease in the number of grain boundaries in the film, leading to limited grain boundary scattering.

As we noted above, co-doping is one of the most promising ways to solve the urgent problem of improving the microstructure of ZnO-based TCO films and, consequently, their electrical characteristics [11,12,13,14,15,37]. To be fully aware of the aspects leading to the improved crystallization and enhanced conductivity of the co-doped ZnO-based TCO films, the role of each introduced impurity has to be considered separately and in combination with each other. With this in mind, and based on the obtained results, we suggest the following model for the improved crystallization process of ZnO thin film co-doped with Al and Ga.

Due to the fact that the Al element is located to the left of Zn in the reactivity series (standard electrochemical potential for Al φ_0_ = −1.7 V), during film growth, many of the Al impurity atoms introduced into the ZnO host tend to oxidize faster than zinc atoms [39]. Thus, the introduced impurity Al atoms, being oxidized, can serve as new crystallization centers, thereby inhibiting the ZnO crystallite growth. At the same time, the more resistant to oxidation Ga metal, located in the reactivity series to the right of zinc (for Ga φ_0_ = −0.56 V), at doping levels not exceeding the limit of Ga solubility in ZnO, is preferably localized to the ZnO host in the form of a substitutional impurity, thus being a more efficient type of real donor impurity for ZnO [25,38,40]. Thus, the introduction of both metal elements (Al and Ga) into the zinc oxide, one located to the left of Zn in the reactivity series and the other to the right, may result in the formation of the preferential separation of the Al and Ga impurities in the thin structure of ZnO film, where Ga impurity is localized to ZnO nanocrystallites, and Al impurity—to the grain boundary space.

In its turn, the observation of some shifts in the (002) ZnO peak position in the low-angle region relative to the position of the lines of the reference ZnO (34.42°) may indicate a stoichiometric deviation in the deposited single-doped and co-doped ZnO films toward oxygen deficiency due to the partial desorption of oxygen from a growing film surface already at a moderate substrate temperature (close to 300 °C) [41]. We believe that oxygen desorption at a substrate temperature of 300 °C and the introduction of both elements in ZnO films can significantly increase the migration length of adatoms on the growth surface [42]. It is known that the crystallinity and electrical properties of ZnO-based TCO thin film can be improved by using slightly reduced conditions during its deposition [43,44]. The Al–Ga phase diagram assumes the presence of a liquid phase at equal molar fractions of Al and Ga close to the temperature of 300 °C. The probability of the formation of this phase increases when growth is carried out under nonstoichiometric growth conditions (oxygen deficiency). This should lead to an improvement in the microstructure of the films due to approaching more equilibrium growth conditions.

Moreover, an increase in the doping level of Al, Ga co-doped ZnO thin films leads to the early coalescence of thin films. A decrease in the thickness of the highly disordered sublayer, which is one of the characteristics of ZnO films deposited via magnetron sputtering, can also contribute to the intensification of ZnO crystallization [32].

## 4. Conclusions

In this work, ZnO-based transparent-conducting thin films were prepared at various substrate temperatures via direct current magnetron sputtering, where high conductivity of the ZnO films was achieved by using both the single-doping method (with Al or Ga) and co-doping method (Al and Ga). The main result was the discovery of the optimal level of Al and Ga impurities content in the sputtered target, at which the co-doped ZnO thin films exhibited improved crystallinity and the accompanying high Hall mobility.

The 150 nm thick ZnO TCO thin films deposited at a substrate temperature of 300 °C by sputtering a target containing 2 at.% each of the Al and Ga impurities demonstrated low resistivity ρ = 3.2 × 10^−4^ Ω·cm due to their higher Hall mobility among other Al or Ga single-doped samples. 

Possible reasons for the improvement in the crystallinity of ZnO films as a result of the simultaneous introduction of Al and Ga impurities were considered.

## Figures and Tables

**Table 1:** Impurity composition of the sputtered ZnO-based ceramic targets and the corresponding abbreviation of the deposited films.

Impurity Content in the Target		Thin Film Sample Abbreviation
at.% Al	at.% Ga	
0	0	ZnO
1	0	1AZO
0	1	1GZO
1	1	1A1GZO
2	0	2AZO
0	2	2GZO
2	2	2A2GZO

**Table 2:** XRD data for intensity *I*, angular position 2θ, and integral breadth β of (002) ZnO peak for the thin films deposited at 300 °C.

Sample	Data for (002) XRD Peak in Thin Films		
	*I*, cps	2θ, deg	β, deg
ZnO	10,248	34.39	0.511
1AZO	5155	34.33	0.572
1GZO	6812	34.21	0.592
1A1GZO	1905	34.31	0.636
2AZO	909	34.28	0.755
2GZO	13,799	34.25	0.546
2A2GZO	27,436	34.24	0.279

## Data Availability

Not applicable.

## Supplementary Materials

The following supporting information can be downloaded at: https://www.mdpi.com/article/10.3390/ma15175862/s1, Figure S1: Transmittance spectra of glasses covered at 300 °C by single-doped (2AZO, 2GZO) and co-doped (1A1GZO, 2A2GZO) thin films. Figure S2: SEM micrographs of 2AZO (**a**), 2GZO (**b**), and 2A2GZO (**c**) thin films deposited at sub-strate temperature of 300 °C.
