# Supplementary material for: On the Effect of the Co-Introduction of Al and Ga Impurities on the Electrical Performance of Transparent Conductive ZnO-Based Thin Films
Source: Materials (Basel). 2022 Aug 25;15(17):5862. doi: 10.3390/ma15175862 (PMC9457261; doi:10.3390/ma15175862)
Supplement: Supplementary file 1 [file materials-15-05862-s001.zip › materials-1852380-supplementary.pdf]

materials

Communication

On the Effect of the Co-Introduction of Al and Ga Impurities on the Electrical Performance of Transparent Conductive ZnO-Based Thin Films

Abil S. Asvarov [1,2,*], Aslan K. Abduev [3], Akhmed K. Akhmedov [1] and Vladimir M. Kanevsky [2]

1 Institute of Physics, Dagestan Research Center of Russian Academy Sciences, Yaragskogo Str., 94, 367015 Makhachkala, Russia
2 Shubnikov Institute of Crystallography, Federal Scientific Research Center "Crystallography and Photonics" of Russian Academy of Sciences, Leninsky Prospect, 59, 119333 Moscow, Russia
3 Basic Department of Nanotechnology and Microsystem Technology, Academy of Engineering, RUDN University, 117198 Moscow, Russia
* Correspondence: abil-as@list.ru

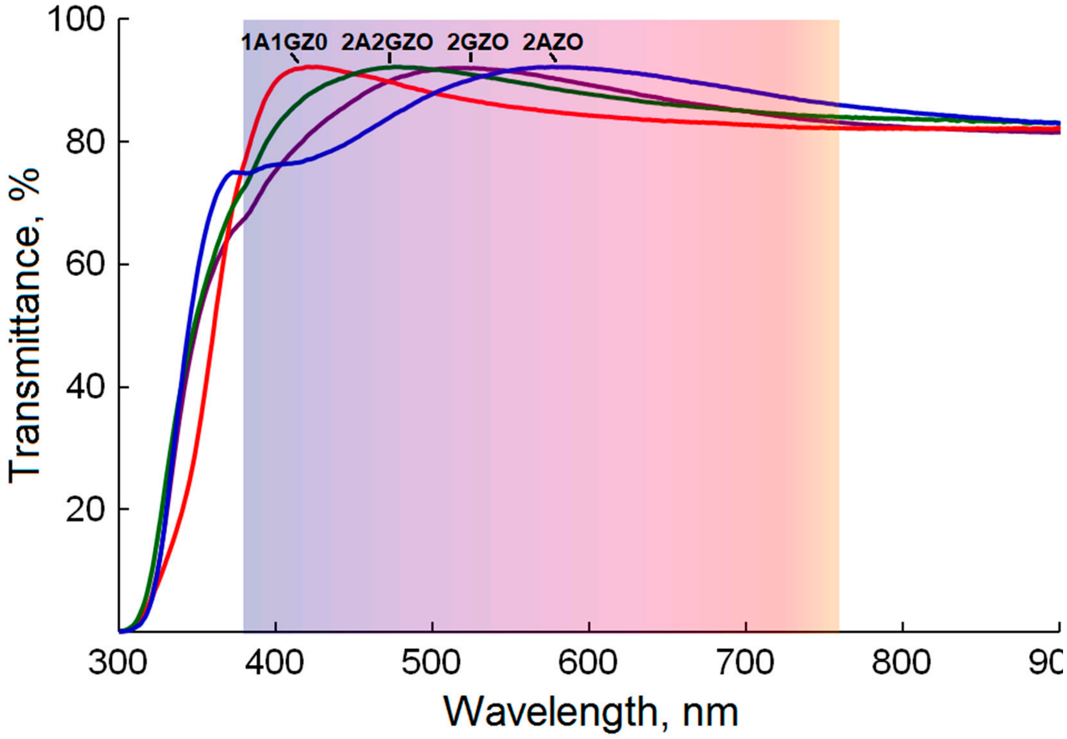

Copyright: © 2022 by the authors. Licensee MDPI, Basel, Switzerland. This article is an open access article distributed under the terms and conditions of the Creative Commons Attribution (CC BY) license (https://creativecommons.org/licenses/by/4.0/).

Figure S1. Transmittance spectra of glasses covered at 300°C by single-doped (2AZO, 2GZO) and co-doped (1A1GZO, 2A2GZO) thin films.

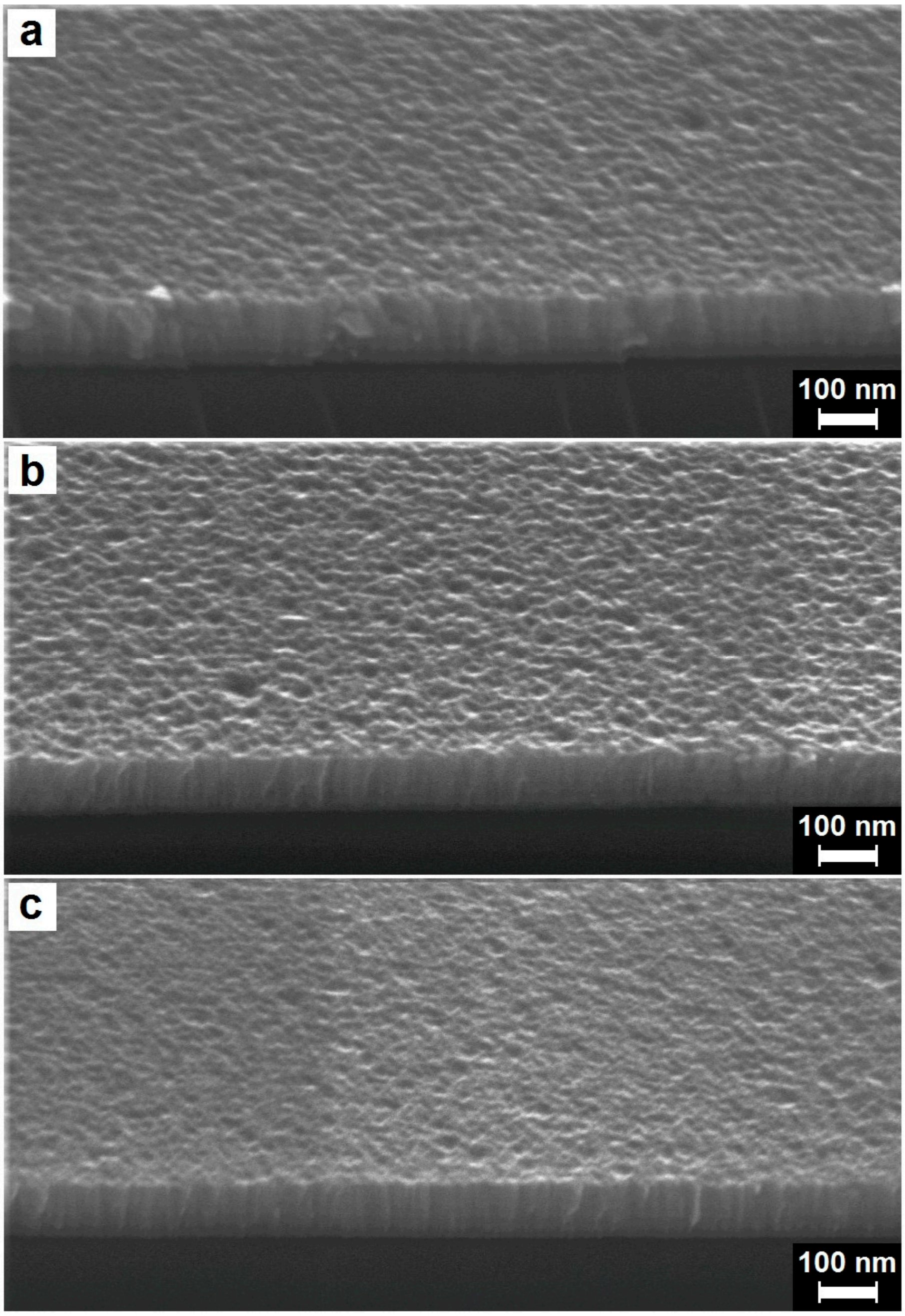

Figure S2. SEM micrographs of 2AZO (a), 2GZO (b), and 2A2GZO (c) thin films deposited at substrate temperature of 300°C.
